# Sex and Gender Differences in Iron Chelation

**DOI:** 10.3390/biomedicines12122885

**Published:** 2024-12-18

**Authors:** Sarah Allegra, Stefano Comità, Antonella Roetto, Silvia De Francia

**Affiliations:** Department of Clinical and Biological Sciences, University of Turin, San Luigi Gonzaga University Hospital, 10043 Orbassano, Italy; stefano.comita@unito.it (S.C.); antonella.roetto@unito.it (A.R.); silvia.defrancia@unito.it (S.D.F.)

**Keywords:** iron overload, deferoxamine, deferiprone, deferasirox, luspatercept, personalized medicine

## Abstract

Background/Objectives: In the absence of physiological mechanisms to excrete excessive iron, the administration of iron chelation therapy is necessary. Age and hormones have an impact on the absorption, distribution, metabolism, and excretion of the medications used to treat iron excess, resulting in notable sex- and gender-related variances. Methods: Here, we aimed to review the literature on sex and gender in iron overload assessment and treatment. Results: The development of iron chelators has shown to be a successful therapy for lowering the body’s iron levels and averting the tissue damage and organ failure that follows. Numerous studies have described how individual factors can impact chelation treatment, potentially impact therapeutic response, and/or result in inadequate chelation or elevated toxicity; however, most of these data have not considered male and female patients as different groups, and particularly, the effect of hormonal variations in women have never been considered. Conclusions: An effective iron chelation treatment should take into account sex and gender differences.

## 1. Introduction

Women and men respond differently to treatments: this mainly depends on physiological, anatomical, and hormonal characteristics [1]. Treatment response is influenced by variations in the pharmacokinetics and pharmacodynamics of therapeutic agents. Even though this has been known since 1932, when the first study on a gender difference in the pharmacology of barbiturates in rats was published, it was not until the end of the 20th century that the significance of gender pharmacology was fully recognized [1,2,3,4,5]. Pharmacokinetics is the study of how a medication moves through our bodies in four stages: absorption, distribution, metabolism, and elimination. These four phases exhibit notable sex-related differences since they are mostly impacted by age and hormones. Pharmacodynamics, on the other hand, investigates the physiological and biochemical effects, as well as the mechanisms of action, of medicinal agents and shows how they affect our bodies. There are numerous differences in pharmacodynamics depending on sex, mainly mediated by hormones, genes, and the environment [6,7,8,9,10,11,12,13]. While, however, the pharmacokinetic differences are simpler to analyze, differences in pharmacodynamics are more difficult to detect [2,14]. However, both should merit a thorough examination of gender differences at the preclinical stage; otherwise, the ensuing clinical stage will be constrained and imprecise [15].

Here, we aimed to review the literature on sex and, if possible, gender in iron overload assessment and treatment.

## 2. Iron Metabolism

Iron is a necessary trace element for many living organisms’ biological functions. Most of the iron content in mammals’ bodies is used for heme synthesis and therefore erythropoiesis, but a modicum is also transported to the peripheral tissues. Here, iron can be used in different ways depending on the needs of the specific cell: it can be brought directly to the mitochondria and other sites of utilization, or it can be stored in a ferritin-bound form. The concentration and localization of iron in mammals’ bodies must be correctly balanced, as a quantitative anomaly in this element, such as iron deficiency, is the cause of IDA (iron deficiency anemia), one the most common globally diffused conditions [16]. Anemia’s cause is multifaceted and complex, with low hemoglobin levels due to iron deficiency accounting for roughly 50% of all cases.

Anemia has a complicated and multifaceted etiology, and iron deficiency accounts for around half of its cases, making it the primary cause of this disease’s burden. According to recent data, 13% of the world population is affected by IDA, which corresponds to about 850 thousand people. Conversely, iron overload can have cytotoxic effects and cause tissue damage. Specifically, siderosis (also named secondary hemochromatosis) and hereditary hemochromatosis are diseases characterized by severe iron overload, with the first mainly being represented by hemoglobinopathy patients with iron overload and the latter by Hfe hemochromatosis, whose frequency is very high in the Caucasian population (at an allelic frequency of 5–10% in populations with a Celtic background) [17,18].

Complex molecular pathways strive to obtain iron homeostasis, characterized by a balance between iron intake from the diet via the duodenal enterocytes, iron usage, and iron recycling via the macrophages from senescent red blood cells, while an iron reserve is predominantly maintained in the hepatocytes [19,20]. Dietary iron is mainly represented by heme iron, in which the metal is part of heme porphyrin, which is found in meat, poultry, and seafood with considerable bioavailability (25–30% of this form is absorbed) and non-heme iron, present in vegetables, of which only 1–10% is absorbed [21]. During digestion, the heme-containing proteins present in meat, hemoglobin and myoglobin, are released in the stomach’s low pH environment, and heme is made available by the action of proteases in the stomach and intestine. Heme enters into the enterocytes through HCP1 and/or two other putative transporters, the Feline leukemia virus subgroup C receptor 2 (FLVCR2) and the Heme carrier protein 1/Proton-coupled folate transporter (HCP1/PCFT). Once it is in the cytoplasm, heme is metabolized by heme oxygenase (HO) and iron is released, joining the labile iron pool (LIP) present in the cytoplasm. The FLVCR1 transmembrane protein can export excess intracellular heme outside the cells to guard them from surplus heme toxicity [22].

**Figure 1:**
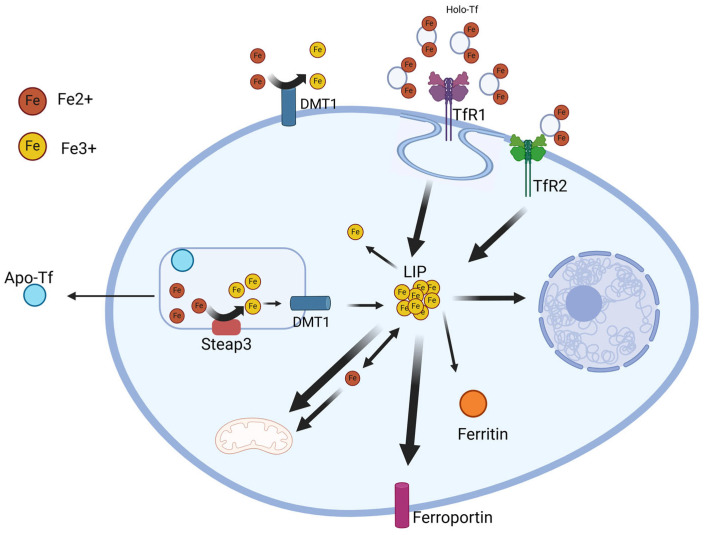
Iron metabolism scheme. Holo-transferrin (Holo-Tf) is created when apo-Tf binds to Fe3+. On the cell surface, Holo-Tf and transferrin receptor 1 (TfR1) form a complex, which is then endocytosed. In the endosome, Fe3+ is liberated from Holo-Tf by a proton pump and converted into Fe2+ by six-transmembrane epithelial antigen of the prostate 3 (Steap3). Divalent metal transporter 1 (DMT1) transports Fe3+ into the cytosol by crossing the endosomal membrane. To begin a new cycle, the apo-Tf released is recycled back to the plasma membrane. The newly acquired iron enters the cytosolic labile iron pool (LIP) and is distributed into various cellular compartments. Unused cellular iron is either exported by ferroportin or retained in ferritin.

Non-heme iron is present in the body in two forms: ferric iron, Fe3+, and ferrous iron, Fe2+. These two forms are employed in different mechanisms, and they are readily converted into their alternative form by oxidases and reductases, a mechanism that is mandatory for iron to be transported across the cell’s membrane. Dietary non-heme iron in reduced by a duodenal-enterocyte-specific reductase called Duodenal Cytochrome B (DCYTB), therefore allowing its passage from the intestinal lumen to the enterocytes via divalent metal transporter 1 (DMT1), where it joins the LIP and, if need be, reaches the bloodstream again via the iron exporter ferroportin 1, present on the basolateral membrane of the duodenal cells [23]. In the bloodstream, Fe2+ is rapidly converted into Fe3+ via the ferroperoxidase Heph (Hephaestin) and is taken up by free Tf (transferrin) for transport to the tissues [24]. Tissue cells expressing TfR1 (transferrin receptor 1) on their plasma membranes introduce the holo-transferrin (Tf-2Fe3+)/Tfr1 complex through clathrin-mediated endocytosis, and the iron is released from Tf in the endosome, reduced by six-transmembrane epithelial antigen of the prostate 3 (Steap3), and released into the cell cytosol via a DMT1-specific isoform. Inside the cell cytoplasm, the LIP can be used to produce iron–sulfur clusters and to synthesize heme in the mitochondria, to be included in iron-containing proteins, or it can be stored by being bound to Ft (ferritin) [25]. If the amount of cellular iron starts to increase, the metal is exported through ferroportin 1. This intracellular iron homeostasis is finely tuned by the so-called IRE/IRP post-transcriptional regulatory system [25]. This described iron metabolism is summarized in Figure 1.

### Hepcidin

**Figure 2:**
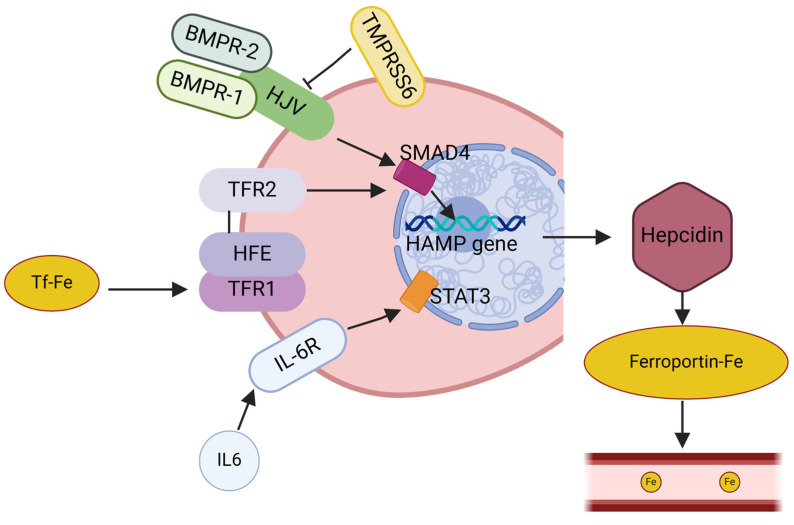
Scheme of hepcidin synthesis. HFE is removed from TFR1 by Tf-Fe2, which then combines with TFR2 and HJV to stimulate hepcidin through bone morphogenetic protein (BMP)/SMAD signaling. Hepcidin is activated by HJV as a co-receptor for the cytokines BMP2 and BMP6. This route involves BMP receptors type I (BMPRI) and type II (BMPRII). To trigger the production of genes controlled by BMP-responsive elements, such as hepcidin, SMAD4 translocate to the nucleus. Matriptase 2, a serine protease encoded by TMPRSS6, cleaves and produces a soluble form of HJV, suppressing BMP/SMAD signaling to hepcidin. Through the interaction of the STAT3 pathway and interleukin-6 (IL6) with its receptor (IL6R), infection and inflammation significantly boost the synthesis of hepcidin.

The master regulator of the body’s iron homeostasis is a small hormone called hepcidin (Hepc), codified by the HAMP gene [26]. Hepcidin is produced mainly by the liver, and its hepatic expression is regulated by the body’s iron demand. In fact, a high demand during iron deficiency reduces HAMP gene expression, while high iron levels stimulate it. Hepcidin binds to the iron exporter ferroportin 1 (Fpn1), and this complex is internalized and degraded, hampering the release of iron absorbed by the enterocytes and its export from stores. Therefore, in the iron deficiency condition, the amount of Hepc is reduced, Fpn1 internalization and degradation is decreased, and more iron can be released from intracellular stores and used, and vice versa. A higher expression of HAMP, and the consequent entrapment of iron in the cells, can also be observed in response to inflammation and infection. This is thought to occur because lowering iron levels during an infection can adjuvate the body’s host defense mechanism as it hampers a pathogen’s metabolism [27]. Hepcidin expression is regulated by several effector proteins that exert a positive or a negative effect on Hepc synthesis [28]. In the physiological conditions of martial deficiency, the amount of Hepc in the plasma and urine is consistent but then rapidly raises in response to increased iron availability. This change is molecularly mediated by a compound of proteins involved in a signal transduction pathway. Briefly, Hfe (hereditary hemochromatosis protein) physiologically binds to TfR1 using certain binding sites that overlap with the Tf/Tfr1-binding sites. In cases of increased iron levels, the Tf saturation rises, Tf-Fe2 forces out Hfe and binds to TfR1, and the complex is internalized. Hfe is disassociated from TfR1 and binds to two other proteins: Hjv (hemojuvelin) and TfR2 (transferrin receptor 2). This complex is able to activate the common BMP-SMAD1/5/8 pathway. A second signal pathway is mediated by BMP2 and BMP6 (bone morphogenetic proteins 2 and 6), whose expression is dependent on iron levels, causing their increased synthesis in cases of iron abundance. These proteins interact with BMP receptors (BMPRs) and hemojuvelin (Hjv), forming a complex that activates the SMAD pathway. Specifically, this consists of inducing the phosphorylation of the SMAD (Small Mother Against Decapentaplegic) regulatory proteins SMAD1, SMAD5, and SMAD8, which, in turn, binds SMAD4. This complex is then translocated to the nucleus, where it induces HAMP gene transcription. On the other hand, hepcidin expression is downregulated in conditions of iron deficiency. The main effector of this negative regulation is Matriptase 2 (or transmembrane serine protease 6 (TMPRSS6)), which senses iron levels and cleaves Hjv, suppressing its action. This blocks the activation of the BMP-SMAD1/5/8 pathway, ending in decreased HAMP gene transcription [25]. Lastly, erythropoietic activity, hypoxia, and inflammation influence hepcidin regulation. Erythroferrone (Erfe) contributes to hepcidin inhibition if erythropoiesis is compromised, while platelet-derived growth factor-BB (PDGF-BB) has the same effect on hepcidin in hypoxic conditions [29]. In inflammatory conditions, interleukin-6 (IL-6) is a proinflammatory cytokine that induces the HAMP promoter [26]. Hepcidin synthesis is summarized in Figure 2.

## 3. Iron Level Measurement

The levels of organic iron deposits should be reflected by serum ferritin. This enables regular monitoring and is measured by blood sample collection. However, a number of conditions, such as infections and inflammations, can affect these outcomes [30].

### 3.1. Liver Biopsy

The most accurate direct technique for determining the presence of hepatic hemosiderosis is biopsy. It makes it possible to assess siderosis quantitatively, including the pattern of metal buildup in the hepatocytes and Kupffer cells and inflammation, fibrosis, and any cirrhosis. According to the Scheuer classification (grades I–IV), hepatic siderosis is quantified using Perls’s method of cytochemical staining for iron. It is also possible to assess the pattern of martial overload at the level of the sinusoids, Kupffer cells, hepatocytes, and the primary structures of the portal space [31].

### 3.2. Ultrasonic Elastography

Numerous techniques for ultrasonic elastography have been created [32]. A low-frequency pulsed excitation that can produce shear waves in the liver tissue is used in transient elastography, which is carried out using FibroScan equipment. The stiffness of the tissue has been linked to the shear wave velocity [33]. There is an outstanding correlation between the stiffness values produced by the FibroScan system and the METAVIR classification (from F0, a healthy liver state, to F4, the most severe stage of fibrosis [34,35]). The ratio of validated measurements to total measurements has been used to determine the rate of successful measurements [33]. These findings are presented as a median value in kPa for all measurements. Fibrosis that is not substantial is indicated by values less than 7.0 kPa [36].

### 3.3. Superconducting Quantum Interference Devices

The physical characteristics of ferritin and hemosiderin—a pigment containing iron that is found in the liver, spleen, and bone marrow and is composed of ferritin molecules and other structural elements—are the foundation of a Superconducting Quantum Interference Device (SQUID). Although the LIC value derived using this non-invasive technique may fully overlap with that from a liver biopsy, it is unable to measure fibrosis and inflammation. A SQUID measures the amount of iron in the spleen and liver [37].

### 3.4. Magnetic Resonance Imaging

Based on nuclear magnetic resonance, magnetic resonance imaging (MRI) is a non-invasive method that is now the gold standard for diagnosing and tracking iron overload disorders [38]. It is sensitive enough to determine the amount and distribution of iron not just in the liver but also in other organs like the heart in the body. There are numerous MRI techniques currently available [39]. A breath-hold echo pulse sequence with multiple echo gradients is used to obtain a succession of pictures with increasing echo times for T2* MRI imaging. These sequences can be used for both the liver and the heart. T2* = 20 ms is the lower limit of normalcy; the lower the T2* number, the greater the chance of a major, and occasionally fatal, cardiac event occurring quickly [40,41]. Normal MRI T2* values, taking the liver into account, are ≥6.3 ms [42].

### 3.5. Serum Iron and Ferritin

It is very well established that the amount of iron varies in the two sexes and according to life period, so much so that in humans, the physiological ranges for serum iron (SI) and ferritin (sFt) are different between men and women (Table 1).

Furthermore, females experience relevant oscillations in iron due to their reproductive metabolism during the menstrual cycle and after the menopause. A recently published paper on a significant cohort of healthy subjects reports that while the serum ferritin values are higher in young males compared to age-matched females, this difference lessens during ageing. Moreover, in women during the perimenopause period, their ferritin levels soar with age, while this increase is milder in postmenopausal conditions [43].

### 3.6. Hepcidin Level Measurements

Soon after the determination of hepcidin as a pivotal regulator of iron metabolism and the discovery of this small peptide in the urine and plasma [44,45], several attempts to determine the amount of Hepc in these biological fluids and to correlate it with a subject’s iron metabolism were undertaken. Nowadays, the most widely used methodologies are enzyme-linked immunosorbent assay (ELISA) and Surface-Enhanced Laser Desorption/Ionization Time-of-Flight Mass Spectrometry (SELDI-TOF-MS). Specifically, using a set ELISA kit, a good correlation between the amount of Hepc in the urine and serum has been determined, as well as serum Ft values. Furthermore, it has been highlighted that the amount of Hepc has a circadian variation that parallels the circadian variation in SI [46]. Lastly, it has been demonstrated in animal models that hepcidin quickly responds to inflammation, irrespective of animals’ anemic conditions [47]. Sex differences in serum hepcidin levels were unraveled in a considerable number of healthy subjects participating in the Nijmegen Biomedical Study [48]. Determining the amount of Hepc in male and female groups stratified by 5-year intervals of age, the amount of Hepc mostly remains constant in males, while the hepcidin concentrations in women mildly increase through menopause (4.1 nM median for women younger than 55 years and 8.5 nM for women 55 years of age and older). Regardless, it must be underlined that the hepcidin concentration in the serum has significant variability among subjects, so the accepted reference ranges are quite wide.

## 4. Animal Models

Concerning the animal models typically used to investigate iron overload and iron chelation, differences related to the sex of the animals have not been taken into account. However, we have decided to briefly list the models used in studying iron overload. Iron overload can be induced in rodent models, like mice or rats, via nutritional manipulation or genetic mutation. High-iron diets or iron compound injections can cause excessive iron buildup in a variety of tissues, simulating iron overload illnesses in humans. These animal models allow for study of the pathophysiology of iron overload and assessments of the effectiveness of iron chelation therapy [49]. Knockout mice, missing the genes involved in iron management and homeostasis, have helped researchers to understand the molecular pathways driving iron overload and create targeted therapeutics [50]. Non-human primates, such as baboons and macaques, additionally can be utilized as models for iron overload and chelation research. These animals have physiological and genetic similarities to humans, making them pertinent to translational study. Non-human primate models give a more precise characterization of iron metabolism, chelation therapy, and potential adverse effects by mimicking human physiology [51]. The zebrafish (Danio rerio) has been developed as a well-known model organism for biomedical research. It has similar metabolic routes to humans and can be manipulated to induce iron overload. Zebrafish models allow researchers to analyze the consequences of iron overload, test prospective chelators, and probe the underlying molecular pathways in an advantageous way and using high amounts [52]. These animal models have certainly brought advantages for studying the pathophysiology of iron overload diseases, evaluating the effectiveness of iron chelation therapy, and understanding the underlying molecular pathways. They enable researchers to explore the impacts of iron overload at several levels, including tissue pathology, gene expression, and metabolic parameters, therefore advancing the development of tailored therapies for iron-related illnesses. Concerning the rodent models discussed above, mutant mice models have been generated to replicate iron overload diseases. Mice with specific mutations in iron metabolism genes, such as Hfe, hepcidin, or transferrin receptors, can be used to explore the molecular processes behind iron excess and chelation [49]. Additionally, high-iron dietary regimens have helped scientists to monitor iron consumption and investigate their effects on long-term iron excess. Dietary models are beneficial because they accurately represent the dietary iron overload reported in certain human groups, such as those with genetic hemochromatosis or heavy iron supplementation. In recent years, genetic approaches such as gene knockout or knockdown have been employed to change different genes related to iron metabolism processes. By altering the genes involved in iron absorption, storage, and transport, these models allow for the investigation of specific molecular targets and processes involved in iron homeostasis and chelation [50]. Scientists can utilize these distinct animal models to explore various aspects of iron overload, such as its origins, processes, development, and potential therapies. These models enable in-depth research into iron metabolism and chelation therapy and the evaluation of new therapeutic methods for addressing iron overload illnesses.

## 5. Chelation Therapy

Iron chelation therapy must be administered when the body lacks the capacity to eliminate excess iron, which causes a negative net iron balance [53,54,55]. While the routine therapy consists of periodical phlebotomies to normalize serum iron parameters for genetically iron-overloaded patients [56], iron chelation is applied to the majority of diseases with secondary iron overload. A chelating reagent binds a metallic atom functioning as a Lewis acid via several coordinating bonds in a chemical reaction known as chelation. The resulting compound’s structure is a fairly stable complex, with the chelator encircling the center atom. A polydentate binder, such as bidentate, tridentate, etc., is frequently used as the chelator. After it is chelated, the metal loses its properties and can be separated from the chelator. In addition to binding and eliminating iron from the body, chelation therapy for beta-thalassemia patients must also attempt to meet other critical requirements [57]: to prevent iron accumulation and the negative effects of its overload, the elimination rate must be equal to or greater than the iron input rate with the transfusion. Therefore, the therapy must allow for flexible dosage and provide 24 h chelation coverage. This means that a molecule with a long half-life is required, and the timing and route of administration (the treatment regimen) must ensure the maximum adherence to therapy. The duration of chelation therapy exposure is critical, and treatment-related side effects must be kept to a minimum, as the number of days for which patients receive the treatment is more significant than the overall dose received during treatment. Currently, deferoxamine, deferiprone, deferasirox (DFX), and luspatercept are the primary medications utilized in iron chelation therapy.

### 5.1. Deferoxamine

Approved in 1970, deferoxamine (Desferal^®^) was the first commercial iron chelator. The Fe–deferoxamine complex is a large hexadentate molecule that binds to iron with great affinity and has a short half-life (20–30 min). It is excreted in the urine and feces [58]. A gradual parenteral infusion lasting eight to twelve hours given five to seven times a week is necessary for deferoxamine ingestion. Adherence to this treatment plan is greatly impacted, and many patients do not fully benefit from it and pass away prematurely. Furthermore, because of its short half-life, deferoxamine cannot provide 24 h chelation coverage [59]. Because it is charged, the deferoxamine–iron chelate struggles to enter and exit cells. This medication eventually causes a number of adverse effects, including skeletal abnormalities, hypoacusis, eye toxicity, delayed growth, and a local response at the site of the infusion [59,60]. It has been determined that treatments that target iron chelation with deferoxamine and the suppression of iron-dependent cell death (ferroptosis) [61] with Edaravone, a medication that is clinically licensed for the treatment of ischemic stroke, are therapeutically promising [62]. Diabetes increases the risk of ferroptosis since it modifies several pathways implicated in the condition. Iron chelation prevented stroke-induced vasoregression, blood–brain barrier breach, and neuronal damage in male diabetic rats [63,64]. Li et al., in a study on the impact of deferoxamine therapy on the poststroke outcomes in female rats with and without diabetes, observed the following sex-related differences [65]: The drug induced deficits in fine motor skills in the male control rats but not in the female control rats, while the working memory deficits at baseline were more pronounced in the female rats than in the male rats, poststroke vascularization was not seen in female diabetic rats as opposed to male diabetic rats, poststroke vasoregression was not seen in female diabetic rats as opposed to male diabetic rats, and deferoxamine decreased the vascular volume and surface area indices in females but increased them in males. On the other hand, in the male diabetic rats, the drug reduced poststroke microglial activation. Remarkably, in the diabetic female rats, deferoxamine enhanced the anti-inflammatory phenotype, suggesting a variety of microglial morphological and functional processes that explain the effects of the therapy in both male and female rats. In the context of diabetes, research on brain microvascular endothelial cells (BMVECs) in both male and female participants has shown that when cells are exposed to high glucose levels as opposed to normal glucose, ferroptosis occurs in response to hypoxia [66]. Iron-responsive element-binding protein 2 (IREB2), a hallmark of ferroptosis, was expressed at notably higher levels in male cells than female cells. Only male cells that were susceptible to the ferroptosis inhibitor Fer-1 showed decreased glutathione peroxidase expression when ferroptosis was induced with erastin. The cytoprotective effects of deferoxamine on male and female BMVECs treated with hemin were also shown in other tests. However, in the hemin-treated cells, the cytoprotective effects of the drug showed minor sex-dependent variations, linked to the expression of glutathione peroxidase and important proinflammatory proteins. Lastly, these provocative in vitro results, when combined, imply that iron chelation may be harmful in non-diabetic circumstances. All things considered, the BMVEC results corroborated the in vivo evidence demonstrating the therapeutic effectiveness of iron chelation in both males and females.

### 5.2. Deferiprone

The Fe–deferiprone complex is not loaded and can therefore readily pass through membranes, enabling quick clearance of iron accumulation from the cells. Deferiprone (Ferriprox^®^), the first oral chelator, is a bidentate chelator (three molecules bonded onto an iron ion), and it was approved in 1987 [58]. Because it is administered orally three times a day and has a half-life of three to four hours, it cannot provide the same 24 h chelation coverage as deferoxamine. Additionally, deferiprone medication has been linked to mild neutropenia, erosive arthritis, rare but severe agranulocytosis, and abdominal pain [59,67,68]. A study on deferiprone-induced agranulocytosis reported that agranulocytosis and neutropenia appeared to be dose-independent and three times more frequent in females than males [69]. Bellanti and colleagues indicated a statistically significant effect of gender on the volume of distribution, which may ultimately affect peak plasma concentrations, in contrast to data from a non-compartmental analysis [70]. Furthermore, adjustments of the deferiprone dose are advised for individuals with decreased creatinine clearance, according to simulated scenarios. For patients with mild, moderate, and severe renal impairment, doses of 60, 40, and 25 mg kg^−1^ are suggested based on creatinine clearance values of 60–89, 30–59, and 15–29 mL min^−1^, respectively. To conclude, their work highlights the need for additional research on the pharmacokinetics of deferiprone in young children and toddlers, for whom pharmacokinetic data do not yet support the current dosing recommendations. Combination therapy with deferiprone and deferoxamine has been investigated for the removal of cardiac iron and the normalization of its storage in the body [71]. Moreover, it has been demonstrated to reverse anomalies in glucose metabolism and enhance gonadal function more successfully, as it is more effective in lowering the body’s total iron burden [72]. Oral glucose tolerance tests revealed considerably lower mean glucose levels in patients that received combo medication. Men and women’s gonadal function and fertility improved, and some patients were able to conceive successfully [72]. According to research on animals, deferiprone is teratogenic and embryotoxic. It is recommended that women of reproductive age receiving deferiprone therapy either refrain from becoming pregnant or switch to a different iron chelator if they intend to become pregnant. Deferiprone should also be avoided by nursing moms [73].

### 5.3. Deferasirox

Deferasirox (DFX) was approved quickly due to the pressing need for a once-daily oral iron chelator that was both safe and effective [74,75]. Two molecules are combined to form a stable complex with an iron ferric atom (Fe3+) to generate DFX, a tridentate iron chelator. ICL670, the lipophilic active molecule, is firmly attached to proteins, particularly albumin. In the clinically relevant range (0.1–0.5 mg/kg/day), it causes a mean net iron excretion of 0.119 mg Fe/Kg body (at a 10 mg/Kg/day DFX dose), 0.329 mg Fe/Kg body (at a 20 mg/Kg/day DFX dose), and 0.445 mg Fe/Kg body (at a 40 mg/Kg/day DFX dose) each day [76]. Its high and specific affinity for Fe3+; oral bioavailability; ease of use in pediatric patients; high efficiency and effectiveness; long half-life (8–16 h), which establishes 24 h chelating coverage; once-daily dosage; flexible therapeutic regimen; and generally good tolerance are the characteristics of DFX chelating. The Food and Drug Administration (FDA) and the European Medicines Agency (EMA) both authorized DFX as a first-line treatment for iron excess associated with blood transfusions in 2005 and 2006, respectively. It can be used in patients with chronic iron overload from either non-transfusion-dependent or transfusion-dependent thalassemia or anemia who are at least two years old. According to the EMA guidelines, DFX treatment should only begin when serum ferritin levels are greater than 1000 µg/L or after the transfusion of at least 100 mL/kg of red blood cells (i.e., at least 20 units for a person weighing 40 kg) [77]. With the exception of people with a greater iron burden (30 mg/kg), the suggested starting daily dose is 20 mg/kg, taken on an empty stomach at least half an hour before meals. Dosage modifications can be made in increments of 5–10 mg/kg/day, up to 40 mg/kg/day, to meet the therapeutic objectives [74]. Water, orange juice, or apple juice can be used to dissolve the pill, and any leftover liquid can be reconstituted into a lesser amount of beverage. DFX should be taken without food at least half an hour before eating [58]. The liver is where DFX is primarily metabolized (glucuronidation), and it is excreted in the feces through hepatobiliary excretion [75,78,79,80,81]. The primary UDP-glucuronyltransferase (UGT) isoform that causes DFX glucuronidation is 1A1 (UGT1A1) [74,82,83,84,85]; in vitro research has revealed the involvement of cytochrome-P450 (CYP) 1A1, 1A2, and, to a lesser extent, 2D6 enzymes [78]. Specifically, UGT converts DFX into the metabolites M3 (acyl glucuronide) and M6 (2-O-glucoronide); CYPs metabolize the remaining 6% of the pro-drug into M1 (5-hydroxy DFX) and M4 (5’-hydroxy DFX), respectively [81]. Multidrug resistance protein 2 (MRP2, also called ABCC2) is primarily responsible for the excretion of DFX and its metabolites in the bile [78], whereas breast cancer resistance protein (BCRP1, also called ABCG2) may have an impact on drug toxicity [82]. In contrast, adequate responders have a trend in ferritin below 1000 ng/mL (calculating the difference between the LIC at the start and end of a study); a declining iron burden in the liver, as shown using MRI or biopsy; and a DFX administration of 30 mg/Kg per day or less. Chirnomas et al. defined inadequate responders as patients with a rising ferritin trend over three consecutive months, at least one measurement higher than 1500 ng/mL, or a rising LIC, as documented using a biopsy or non-invasively and on a dose of more than 30 mg/Kg per day of DFX [86]. We established a threshold for an effective DFX concentration at the end of the dosing interval (Cthrough) of 20,000 ng/mL and area under the curve (AUC) concentrations for efficacy (360 μg/mL/h) and non-response (250 μg/mL/h) cut-offs based on Chirnomas et al.’s definition of efficacy [87,88]. Adults and children with various forms of chronic anemia tolerate DFX well. The clinical safety profile of DFX in patients of all ages, including those under two years old, has been characterized and documented by phase II and III investigations [89]. Its most frequent side effects are mild, moderate, or severe rashes and gastrointestinal problems (diarrhea, stomach pain, nausea, and vomiting). A non-progressive rise in blood creatinine levels proportionate to the chelating dose is seen in one-third of patients receiving treatment; this rise resolves on its own and falls as the medication dosage is decreased. Acute renal injury (including in pediatric patients), hepatic toxicity, cytopenia (agranulocytosis, neutropenia, and thrombocytopenia), and negative effects on hearing and vision have been documented in recent research. There appears to be no correlation between the length of treatment and a rise in side effects [57]. Considering the drug pharmacokinetics, De Francia and colleagues observed difference between sexes: females had a mean DFX Cthrough of 16.79 ± 17.46 μg/mL, which was higher than the value reported for males, at 12.90 ± 13.49 μg/mL [84]. In contrast to previous authors, Mattioli et al. indicated that gender has no effect on plasma concentrations, but we did discover a trend toward an inverse relationship between DFX and age [90]. Given that the drop in DFX clearance may be more than 20%, close monitoring of the liver and kidney functions in DFX-treated patients is necessary [91]. This decrease could put kids at a higher risk of drug-induced toxicities, which would seriously impair the activity and function of already damaged organs. Adding a body-weight-based allometric scaling exponent of 0.75 to population pharmacokinetic models may improve the prediction of drug clearance by accounting for differences in organ functioning [92]. This approach has been questioned by several studies, however, as the exponent value in children can range from 0.6 to 1.11 [93]. In infants, toddlers, and kids, for example, such variability could be between 0.50 and 1.20 [91].

### 5.4. Luspatercept

Luspatercept, a recombinant fusion protein and an erythroid maturation agent, is the most recently approved drug for treating individuals with transfusion-dependent anemia brought on by lower-risk myelodysplastic syndrome or β-thalassemia. It binds and inhibits some transforming growth factor-β superfamily ligands, such as growth differentiation factor 11. This interferes with Smad2/3 signaling, which is markedly increased in disease situations where erythropoiesis is unsuccessful [92]. Therefore, through the differentiation of late-stage erythroid precursors, or normoblasts, luspatercept stimulates erythroid maturation in the bone marrow [92]. Moreover, it promotes erythropoiesis and reduced Smad2/3 signaling [94,95]. Over a dose range of 0.125–1.75 mg/kg, luspatercept demonstrated linear pharmacokinetics, with first-order absorption and elimination [92,96]. The maximum drug concentration and AUC in the serum increased roughly proportionately with increasing dose [92]. The median time to reach the maximum drug concentration was around seven days, and after three doses (a total of nine weeks), a steady state was attained, with an accumulation ratio of about 1.5 [92]. In patients with myelodysplastic syndromes, its mean half-life in their serum was approximately 13 days, while in those with β-thalassemia, it was around 11 days [92]. There was a mean apparent total clearance of 0.52 L/day and 0.44 L/day. Luspatercept is eliminated in the urine since its molecular mass is greater than the glomerular filtration size exclusion threshold, and it is broken down into amino acids in a variety of tissues [92]. Luspatercept’s pharmacokinetics does not seem to be significantly impacted by patient age, sex, race, β-thalassemia genotype or ring sideroblast status, splenectomy status, mild to moderate kidney impairment, or specific baseline laboratory values [92,96]. Body weight is the only clinical measure that coincides with the volume of distribution and clearance, which supports a body-weight-based dosing strategy [96]. One pediatric study aims to evaluate the pharmacokinetic profile in younger patients because this may be a problem with extreme body weights in particular in the pediatric population [97]. In healthy postmenopausal women, luspatercept enhanced their hematological parameters (increased RBC, hemoglobin, and hematocrit levels) [98]. Thirty-two healthy postmenopausal women participated in a phase I trial in which they were given two subcutaneous doses of either luspatercept (0.0625–0.25 mg/kg) or a placebo (3:1 randomization) separated by two weeks. Curiously, this study cohort was selected because osteoporosis may be well treated with luspatercept and other comparable compounds (such as sotatercept). Furthermore, it was unclear how the hypothalamic–pituitary–gonadal axis’s activin signaling might be inhibited [99]. Assessing the safety, tolerability, pharmacokinetics, and pharmacodynamic consequences of increasing the luspatercept dosage levels was the aim of this experiment. Three cohorts of 8 patients each were created from 24 patients, and they were given three increasing doses (0.0625, 0.125, and 0.25 mg/kg). During the research, there were no serious side effects, and luspatercept was well tolerated. Beginning seven days after the injection and continuing for many weeks after therapy, a dose-dependent rise in the hemoglobin concentrations was noted in the treated patients. A total of 83.3% of participants in the highest dose group (0.25 mg/kg) experienced a rise in hemoglobin of ≥1.0 g/dl. Notably, luspatercept’s action may seriously harm embryonic development, as demonstrated by animal studies; for this reason, it must be avoided during pregnancy. This drug should be avoided during breastfeeding because it is secreted into the milk of nursing rats and is passed through the placenta of pregnant rats and rabbits [100]. This is likely to happen in humans as well [101,102]. The use of luspatercept in men and women of reproductive age must be accompanied by appropriate contraceptive counselling and pregnancy planning for these reasons, which make it completely inappropriate during pregnancy.

## 6. Conclusions

Iron levels differ between the sexes and according to life stage; additionally, because of the menstrual cycle and menopause, females experience relevant oscillations in iron. Young males have higher serum ferritin levels than age-matched girls, although this difference decreases with age. Ferritin levels rise with age in women going through the perimenopause, but this rise is less pronounced in postmenopausal women. Also, serum hepcidin levels differ by sex: Hepc is found to mostly be constant in males, whereas its concentrations slightly rise throughout menopause [48]. Differences in the animals’ sexes have not been considered in the animal models commonly used to study iron overload and iron chelation. Considering deferoxamine, in male diabetic rats, iron chelation prevented stroke-induced vasoregression, blood–brain barrier disruption, and neuronal injury [63,64]. Sex-related variations were also reported when examining the effects of deferoxamine medication on the poststroke outcomes in female rats with and without diabetes [65]. Deferoxamine’s cytoprotective effects in hemin-treated cells displayed slight sex-dependent differences, associated with the expression of key proinflammatory proteins and glutathione peroxidase [66]. Deferiprone-induced agranulocytosis appears to be three times more frequent in females than males [69], and a significant effect of gender on the volume of distribution has been reported [70]. DFX’s Cthrough was higher in females and in younger patients [84,90]. Luspatercept’s pharmacokinetics seems to not be impacted by sex [92,96], but luspatercept’s action may seriously harm embryonic development [100].

Patients with sickle cell disease, thalassemia syndrome, and myelodysplastic syndromes who need daily transfusions may suffer from chronic iron overload. One of siderosis’s harmful impacts is increasing destruction of the organs and tissues, which impairs their ability to operate. The development of iron chelators has been shown to be a successful therapy for lowering the body’s iron levels and averting the tissue damage and organ failure that follows. Numerous studies have described how individual factors can impact chelation treatment, potentially impact therapeutic response, and/or result in inadequate chelation or elevated toxicity; however, most of these data have not considered male and female patients as different groups, and particularly, the effect of hormonal variations in women has never been considered. Moreover, gender differences in enrolled patients should be highlighted to track drug levels and maximize and ascertain the best dosage for every patient.

## Figures and Tables

**Table 1:** Serum iron levels in adult males and females, newborns, and children.

	Serum Iron (mcg/dL)	Serum Ferritin (ng/mL)
Adul males	65–176	12–300
Adult females	50–170	12–150
Newborns	100–250	25–200
Children (6 m–15 y)	50–250	7–140
